# Real-Time Semantic Segmentation for Fisheye Urban Driving Images Based on ERFNet †

**DOI:** 10.3390/s19030503

**Published:** 2019-01-25

**Authors:** Álvaro Sáez, Luis M. Bergasa, Elena López-Guillén, Eduardo Romera, Miguel Tradacete, Carlos Gómez-Huélamo, Javier del Egido

**Affiliations:** Electronics Department, University of Alcalá, Campus Universitario, 28805 Alcalá de Henares, Spain; alvaro.saezc@edu.uah.es (Á.S.); elena.lopezg@uah.es (E.L.-G.); eduardo.romera@edu.uah.es (E.R.); miguel.tradacete@edu.uah.es (M.T.); carlos.gomezh@edu.uah.es (C.G.-H.); javier.egido@edu.uah.es (J.d.E.)

**Keywords:** fisheye, intelligent vehicle, CNN, deep learning, distortion

## Abstract

The interest in fisheye cameras has recently risen in the autonomous vehicles field, as they are able to reduce the complexity of perception systems while improving the management of dangerous driving situations. However, the strong distortion inherent to these cameras makes the usage of conventional computer vision algorithms difficult and has prevented the development of these devices. This paper presents a methodology that provides real-time semantic segmentation on fisheye cameras leveraging only synthetic images. Furthermore, we propose some Convolutional Neural Networks(CNN) architectures based on Efficient Residual Factorized Network(ERFNet) that demonstrate notable skills handling distortion and a new training strategy that improves the segmentation on the image borders. Our proposals are compared to similar state-of-the-art works showing an outstanding performance and tested in an unknown real world scenario using a fisheye camera integrated in an open-source autonomous electric car, showing a high domain adaptation capability.

## 1. Introduction

The most critical task for autonomous vehicles is understanding their surroundings. A good real-time scene-comprehension is vital to a vehicle so it can drive in an unknown environment in a safe way. The semantic segmentation task proposes a solution for this challenge based on image pixel-level classification in multiple semantic categories such as vehicles, pedestrians, traffic signals, etc., satisfying most of the vehicle needs in a unified way [1].

The remarkable success of semantic segmentation solutions during the last few years has been closely related to the breakthrough of deep learning methods, which have proven to widely outperform previous state-of-the-art machine learning techniques [2,3]. Among these techniques, the success of Convolutional Neural Networks (CNNs) has been pushed by the development of excellent open-source deep learning frameworks [4,5], by the progression of specific computational hardware such as Graphics Processing Units (GPUs), and by the appearance of large-scale training datasets [6,7].

The comprehension of a vehicle’s surroundings becomes even more challenging in complex environments such as urban traffic scenes, where the behavior of dynamic traffic participants like pedestrians or vehicles is unpredictable, or specific situations such as intersections or roundabouts that require big volumes of information to be adequately handled. Accordingly, a full real-time perception of the scene is a compulsory need for autonomous vehicles. Different sensors can be used in order to cover this need as cameras, LiDAR, radar, ultrasound, etc. Cameras clearly stand out among other solutions as they are able to generate real-time high-level semantic information while remaining easy to manage, cheap and present low power consumption.

However, the limited field of view of traditional cameras complicates the management of complex environments since cameras are expected to cover the 360${}^{\circ }$ surroundings. The number of devices that compose the perception system is a critical parameter to be optimized, given that a high number of cameras involve high processing times and the fulfillment of a set of hard tasks such as sensor calibration, synchronization and data-fusion.

Fisheye cameras have started to play an increasingly important role in autonomous vehicles because of their ultra-wide field of view. These devices allow for acquiring more scene information using only a sensor at the cost of radial distortion in the images. With fields of view higher than 180 degrees, only two of these cameras are theoretically needed to cover the all of the vehicle’s surroundings. In addition, current autonomous vehicles are betting on redundant and robust perception systems. Fisheye cameras can clearly help in the achievement of these objectives in order to reach safe and reliable driving.

Despite the discussed advantages, distortion associated with these cameras prevents the use of standard computer vision algorithms on the acquired images, making the integration into autonomous vehicles difficult. Furthermore, the application of deep learning techniques to these kinds of images presents many problems such as the lack of large-scale annotated datasets or the management of the distortion, which has caused that only some few works of the state of the art have focused on adapting current semantic segmentation methods to fisheye cameras.

This paper is an extension of our previous conference publication [8]. This work proposes robust deep learning techniques and some CNN architectures able to handle fisheye distortion correctly and that allows real-time fisheye semantic segmentation without the need for using pixel-level hand-annotated images. Moreover, our proposals have been validated in an open-source dataset such as CityScapes and an additional dataset obtained from our open-source autonomous electric car.

This paper is organized as follows: Section 2 examines previous related works. Section 3 introduces the generation of a specific fisheye dataset and some fisheye data augmentation techniques. Section 4 and Section 5 present our CNN architecture proposals based on Efficient Residual Factorized Network (ERFNet), the training strategy and the performed experiments. Finally, Section 6 presents some qualitative results for a real autonomous vehicle.

## 2. Related Work

The main issue with fisheye cameras is how to correctly handle distortion. Distortion is heterogeneous over the different fisheye image areas [9], being a function of both the radial angle and the distance between the principal point of the camera and the image points of the detected objects. This adds complexity to the training of CNNs, as they are forced to learn complicated features that allow the detection of objects with changing appearances depending on their position in the image in order to perform an accurate detection.

An initial approach to deal with the problem is the undistortion of the captured images in order to apply traditional vision techniques [10]. In [11], an end-to-end multi-context collaborative deep network that leveraged semantic information was used to remove distortion from single fisheye images achieving an outstanding performance but with an inadmissible processing time for real-time tasks.

Authors in [12] successfully used a region based CNN (R-CNN) to perform multi-class object detection on panoramic images that were constructed with three fisheye images. The distortion was corrected using a simple and fast approach based on longitude-latitude projection, as correction accuracy was not considered a key issue for object detection.

**Figure 1:**
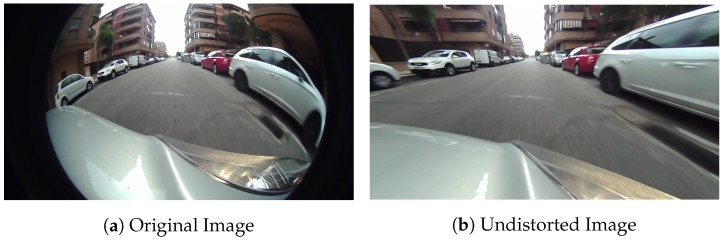
Example of fisheye image undistortion.

Nevertheless, none of the previous works achieved a good quality corrected image in a reasonable processing time as the image undistortion process has several difficulties: the strong dependency on intrinsic camera calibration parameters, the high consumption of computational resources that penalizes real-time processes and, finally, a remarkable loss of image quality, leading to information loss all over the image, but especially in the boundaries as shown in Figure 1. These regions are critical, as they gather a big part of the scene information. To deal with this problem, in [13], a CNN-based preprocessing stage and a multi-frame-based view transformation were proposed and applied in an Around View Monitor system (AVM). However, this approach uses separated CNN frameworks for image enhancement and up-scaling and hole filling method can be improved.

These inconveniences have caused the sprouting up of other approaches that try to adapt existing image processing techniques to work with the distorted images directly instead of the opposite. The lack of available large-scale annotated datasets for non-conventional camera images, like fisheye, has forced the generation of synthetic datasets with additional fisheye distortion leveraging existing ones like CityScapes [7]. In [14], the ETH Pedestrian Benchmark [15] and a spherical perspective imaging model were used to generate a fisheye dataset to allow pedestrian detection with ultra-wide Field Of View (FOV) cameras using a Deformable Part Model (DPM) [16]. In [17], the perspective projection equation of equidistant fisheye camera was used to transform CityScapes images in a new distorted dataset using a mathematical remapping relationship. In [18], the same technique was used combined with additional images generated with a SYNTHIA simulator [19].

The most relevant features to be learned in the CNN learning process are the appearance of the detected objects, their shape and their contextual information [20]. Previous works identified that fisheye distortion penalizes the first two points, while the third one becomes especially important as the appearance of the objects becomes closely related to their position in the images.

Multiple ideas have been proposed to incorporate more context information in order to improve the results of the classification task. Most works have focused on obtaining wide receptive fields to capture valuable information. This can be achieved by including down-sampling stages followed by a set of convolutional layers. However, the down-sampling operation implies the reduction of the feature maps’ scale and, hence, the loss of information. In [21], dilated convolution or Atrous convolution were proposed to enlarge the receptive field of filters without reducing the resolution nor increasing the number of parameters by adding a fixed separation between kernel elements. Deformable convolutions [22] introduce a similar approach where 2D offsets were added to the kernel sampling locations expanding the receptive field of convolutions and improving the ability of modelling geometric transformations [18] but markedly augmenting the number of the network parameters. In order to avoid the increase of the number of parameters, handcrafted structures, like the pyramidal parsing module [23], have been proposed.

In conclusion, multiple ideas are currently proposed to improve CNN performance on fisheye images and handle fisheye distortion correctly, but most of them are not able to achieve real-time semantic segmentation on real fisheye images without resorting to manually annotated images during training.

## 3. Fisheye Synthetic Dataset and Data Augmentation

The generation of large-scale datasets involves expensive and time-consuming tasks such as data acquisition and the corresponding data annotation. Semantic segmentation tasks require pixel-wise annotations, which makes labeling extremely difficult for this kind of images. As we advanced in our precious publication [8], our approach consists of taking advantage of public annotated datasets applying distortions models over RGB and labeled images (ground truth) in order to shortcut the hard manual labelling task. Therefore, we have developed a synthetic dataset from CityScapes using a generic fisheye camera model to add artificial distortion to the images.

### 3.1. Synthetic Fisheye Dataset

Conventional pinhole cameras have a limited field of view defined by their imaging projection, according to the following Equation (1):

(1) $$\rho_{pinhole}=ftan\left(\theta \right),$$

where ρ is the distance between the image point and the camera principal point, *f* is the focal length of the camera and θ is the angle between the incoming light ray and the image principal axis.

In the case of fisheye camera modelling, there are several mathematical models that can be used to design fisheye lenses. Among them, the most widespread one is the equidistant fisheye, which is described by Equation (2):

(2) $$\rho_{equidistance}=f\theta .$$

**Figure 2:**
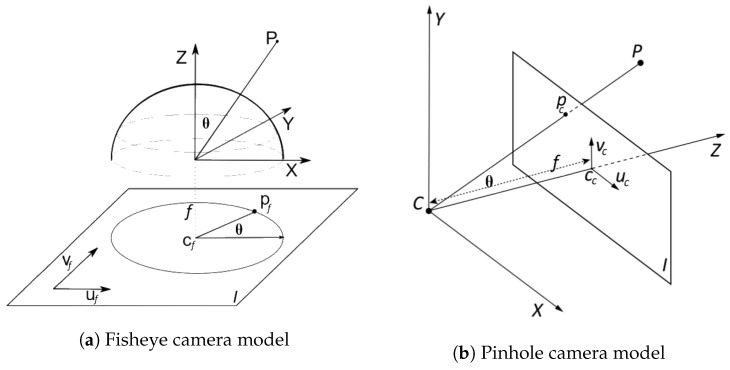
Comparative between camera models.

Using the previous equations, a remapping can be defined between the pixels on a conventional image ($p_{c}=\left(u_{c},v_{c}\right)$) and its analogous pixels on a synthetic fisheye image ($p_{f}=\left(u_{f},v_{f}\right)$), depending only on the *f* parameter, which determines the level of added distortion as shown on Equation (3):

(3) $$d_{c}=ftan\left(d_{f}/f\right),$$

where $d_{c}=\sqrt{{\left(u_{c}-u_{cu}\right)}^{2}+{\left(v_{c}-v_{cv}\right)}^{2}}$ measures the distance between a single pixel ($p_{c}=\left(u_{c},v_{c}\right)$) and the principal point ($c_{c}=\left(u_{cu},v_{cv}\right)$) for the conventional image, and $d_{f}=\sqrt{{\left(u_{f}-u_{fu}\right)}^{2}+{\left(v_{f}-u_{fv}\right)}^{2}}$ represents the equivalent distance for the fisheye image. A comparison between these two camera models is represented in Figure 2.

**Figure 3:**
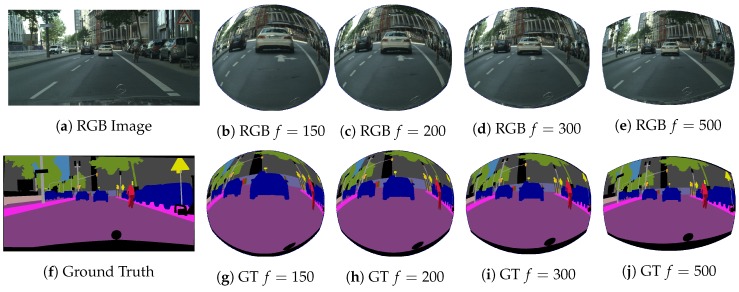
Example of synthetic images and ground truth with different distortions.

Leveraging the previous equation and the CityScapes dataset, a new collection of synthetic distorted images was produced to carry out the training of CNNs with fisheye images representing urban scenes. CityScapes is an optimal dataset for autonomous driving applications, as it is focused on urban scene understanding. It provides 5000 dense pixel-wise annotated images separated into three different subsets (2975 for training, 500 for validation and 1525 for test) and, additionally, another 20,000 with coarse annotations from 27 European cities with 19 classes for evaluation. In our case, only the fine full dataset, including training and validation subsets images, for both RGB and annotated images, were transformed. Original images were resized to 640 × 576, using bi-linear interpolation for RGB images and nearest-neighbor for label images in order to adapt them to our CNN architecture. Some examples of the final synthetic dataset can be seen in Figure 3.

### 3.2. Fisheye Data Augmentation

The features learned by a CNN during training rely mostly on the specific images used during this process. Therefore, these features are limited to the domains in which these images were acquired and should have the property to generalize to different domains. However, achieving robustness in other domains is not an easy task, and deep networks are often prone to overfitting even with thousands of training images. This difficulty is even greater when synthetic images are employed during training, given that appearance in synthetic images is less rich and varied than in real images.

In order to obtain general features, data diversity must be high, due to the huge, different patterns CNNs are forced to learn to be able to distinguish between multiple categories in changing detection conditions. Data augmentation aims to enlarge the training datasets, preserving the available labels by applying different transformations.

For the semantic segmentation task, numerous techniques are typically applied such as geometric augmentations (translations, rotations, horizontal flips, etc.), texture augmentations (color jittering, changes in brightness, contrast, etc.) [24] or specific transformations. For instance, authors in [17] proposed an augmentation technique specifically oriented for fisheye images named zoom augmentation. This data augmentation claimed the use of images with various distortions during training by adopting different fixed values for the *f* parameter from Equation (3) aiming to obtain better generalization abilities. In our previous publication [8], we proposed a modification of this method employing randomly chosen distortions, eliminating the selection process of the fixed distortions and achieving an equal performance.

## 4. CNN Architecture and Training

The demanding needs of real-time applications have boosted the development of efficient network architectures, leaving behind large deep architectures that achieved outstanding performances at the expense of the consumption of computational resources, using different ideas such as Conditional Random Fields (CRFs) [21], residual layers [25] or dilated convolutions [26]. Initial approaches were able to reach real-time semantic segmentation by strongly reducing the number of network parameters, but obtaining poor performances [27].

Our previous proposal ERFNet [28] achieved a remarkable trade-off between efficiency and accuracy. The network has an encoder–decoder structure, like other efficient CNNs such as Enet [29] or SegNet [30], but demonstrates a notable performance due to the use of non-bottleneck residual layers. The use of these layers is more unusual than the bottleneck layers due to efficiency reasons, but non-bottleneck layers have exposed performance improvements in certain shallow architectures like ResNet. However, ERFNet proposes a redesign of these layers using factorized (1D) kernels to build the residual blocks, in order to reduce computation and achieve an efficient architecture while keeping an equivalent performance to the non-bottleneck layers.

**Figure 4:**
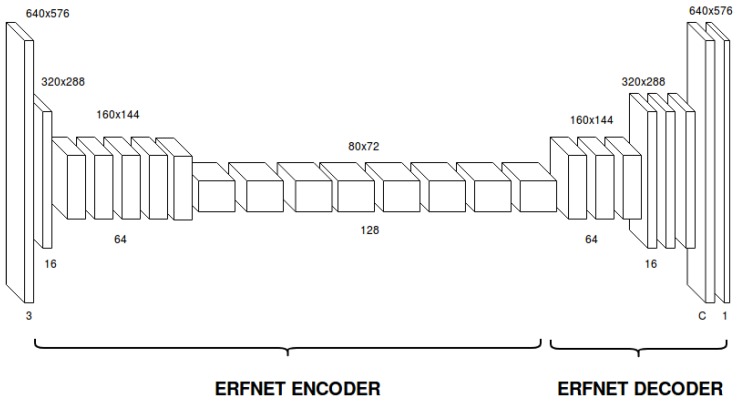
ERFNet baseline architecure.

We adopt ERFNet as our baseline CNN architecture. The basic architecture of ERFNet is presented in Figure 4. Encoders and decoders are both built by stacking 1D-non-bottleneck layers in a sequential way. The encoder module consists of a reduced number of layers including three downsampling blocks and convolutional stages. Encoder is meant to take input images and “encode” them into deep features that represent activations to different image classes. Obtaining good features at this point is essential to produce good classification results. We include three downsampler blocks to perform 8× downsampling in total, which was selected to optimize the trade-off between low-res features (which is more efficient and includes more context) and high-res features (which has better feature localization at the pixel level but is more computationally expensive). In addition, we include dilated convolutions in some of the encoder’s blocks to effectively increase gathering of context without affecting efficiency or resolution.

The decoder module includes upsampling and convolutional layers and a final classification log–softmax loss layer. The decoder stage is meant to preprocess encoded features up to the input’s resolution and provide the final probabilities for each of the trained classes. Thus, the final layer is a volume with a number of slices equal to the number of classes, where each slice contains the per-pixel probabilities of that class. In order to take the predicted (or most probable class), the argmax of this volume is calculated. Many networks use a large decoder, but we chose a relatively small one because the decoder is only meant to upsample features and convert to probabilities, without affecting much to the extraction of good features. Therefore, the encoder does most of the feature extraction job and our light decoder transforms these representations into meaningful outputs in the shape of probabilities.

**Figure 5:**
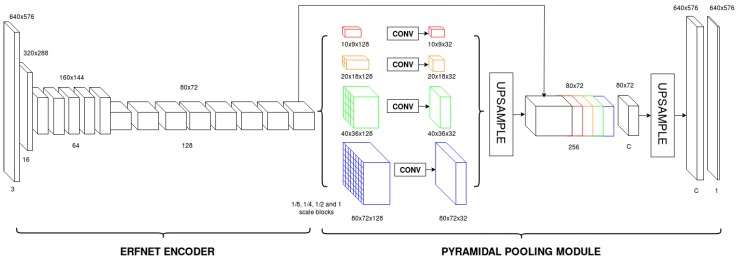
Diagram that depicts the proposed segmentation CNN (ERFNetPSP). Volumes correspond to the feature maps produced by each layer for an example input of 640 × 576.

Considering the significance of context information in fisheye images, we also study the use of an alternative architecture, consisting of replacing the original ERFNet decoder by a handcrafted pyramidal pooling module [31]. Four different pyramid levels are used in the module, including 1/8, 1/4, 1/2 and 1 scale blocks, followed by an upsampling stage and the final log–softmax classification layer. The scheme for this second network is shown in Figure 5 and the layer disposal in Table 1.

The training of both architectures (baseline and modified) is divided into two different stages: on the first one, the encoder module is trained individually using downsampled annotations as ground truth during 90 epochs with a batch size of 6. For the second one, the complete architecture (including the decoder or the pyramidal module) are trained together to produce end-to-end semantic segmentation for another 90 epochs.

The Adam optimization of Stochastic Gradient Descent is used, starting with a learning rate of $5\times 10^{-4}$, which is exponentially decreased on each epoch, and including a weight decay of $1\times 10^{-4}$ for regularization. We employ the class weighing technique introduced in [29]$w_{class}=\frac{1}{ln(c+p_{class})}$ fixing *c* = 10 during the entire training.

**Figure 6:**
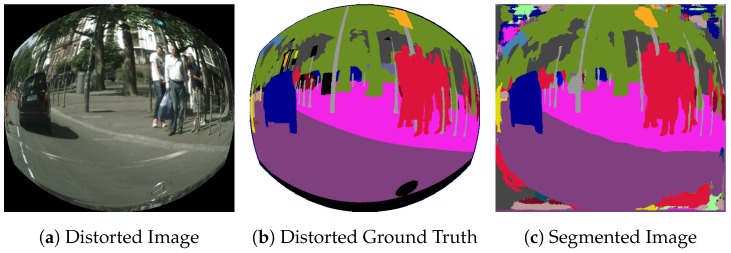
Example of heterogeneous segmentation on the borders.

The black corners in Figure 6a are characteristic of fisheye images. These regions appear on the syntheticly distorted images and on their associated ground truth, as a consequence of the pixel remapping process as shown in Figure 6a. However, the pixels included on those regions are ignored during both training and evaluation. Figure 6c shows an example of segmentation of a synthetic fisheye image.

As it can be seen, these regions present a very heterogeneous segmentation that harms the context information on the borders of the useful parts of the image, which are essential because they contain an important area of the total FOV of the camera. Context information is the most determinant feature for the segmentation of the image borders, due to the strong distortion they present. As a consequence, the performance of the CNN in this area is clearly degraded.

In order to preserve this context information and improve the segmentation on the borders, a new training strategy is proposed. We identify those areas a priori and add an additional 20th class to represent them during training.

## 5. Experiments

For the validation of the proposed architectures, data augmentation and training strategies, different experiments are presented. The first one evaluates our data augmentation strategy and the second one studies the performance of the different architectures compared to other proposals of the state of the art.

To prove the benefits of our data augmentation proposal, a comparative experiment with other approaches of the state of the art was carried out. For the fixed zoom augmentation, three datasets were generated as in [17] with $f_{0}=159$, $f_{1}=96$ and $f_{2}=242$, respectively. For the random zoom-augmentation, the focal length values were randomly changed following a Gaussian distribution, generating five distorted images for each training image as we did in [8]. Additional data augmentation including color jittering (randomly modifying brightness, saturation and contrast to develop a more robust training to light changes), random-cropping (to prepare the CNN for scale and aspect-ratio and scale changes), mirroring, rotations (between 0 and 90 degrees) and arbitrary 0–2 pixels translations was carried out (full data augmentation).

Table 2 presents the experimental results. The first three lines correspond to other state-of-the-art works. Dilation 10 [26] includes dilated convolutions to improve the aggregation of information. ResNet-26 presents the score for a modified 26 layer ResNet with bottleneck blocks and dilated convolutions [17]. OPPNet [17] is composed of a dilated fully convolutional feature extractor block followed by an overlapping pyramid pooling module which analyzes the images at different scales aiming to obtain more context information.

According to Table 2 results, our ERFNet proposals outperform previous state-of-the-art work even without data augmentation. Using ImageNet pre-training improves performance regarding to basic training. The three data augmentation techniques improve both basic and pretrained performances, showing the importance of data diversity. Random and fixed zoom-augmentation provide similar results for both architectures, the random augmentation being more beneficial for the ERFNetPSP and the fixed one for the basic ERFNet architecture. The application of additional data augmentation techniques improves the final results even more, reaching 58.3% and 59.3%, respectively. Both networks clearly stand out in front of previous works, exceeding by 4.8 and 3.8% the previous best score (OPPNet).

In a second experiment, an alternative synthetic dataset with a lower distortion (f=240) was generated. The two proposed architectures were trained without any data augmentation, tested on the validation subset and compared to other state-of-the-art results in the same conditions for fair comparison. Training with an additional class proposed on Section 4 was also tested (ERFNet20), in order to study its benefits.

The comparison includes a set of network models derived from ERFNet [18]. The original ERFNet was re-implemented in MXNet [5] with additional batch normalization layers after each convolutional layer and with 2 × 2 kernels with a stride of 2 on the deconvolution layers (ERFNetMx). Additionally, two extra models were proposed incorporating restricted deformable convolutions (RDCNet), which use a reduced number of parameters, and factorized restricted deformable convolutions (FRDCNet), which can be implemented using 1D kernels.

Furthermore, some additional CNNs were re-implemented in Pytorch to widen the comparative including: a modified PSPNet [31] built by a ResNet-101 with deformable convolutions as the feature extraction block followed by a pyramid pooling module. A modified DRN-D-54 following the proposal of Dilated Residual Networks [32] and including dilated convolutions [26] and SegNet [30], which is able to provide real-time semantic segmentation at the expense of a loss of performance. Finally, ERFNet20 shows the score for the ERFNet training with an additional class to correctly identify the borders of the images. ERFNet and ERFNetPSP models were trained in two stages, as in the the previous experiment, and the rest of CNNs were trained during 200 epochs, using the proposed parameters for them in their respective publications. Results of this experiment are listed in Table 3 and Table 4.

**Figure 7:**
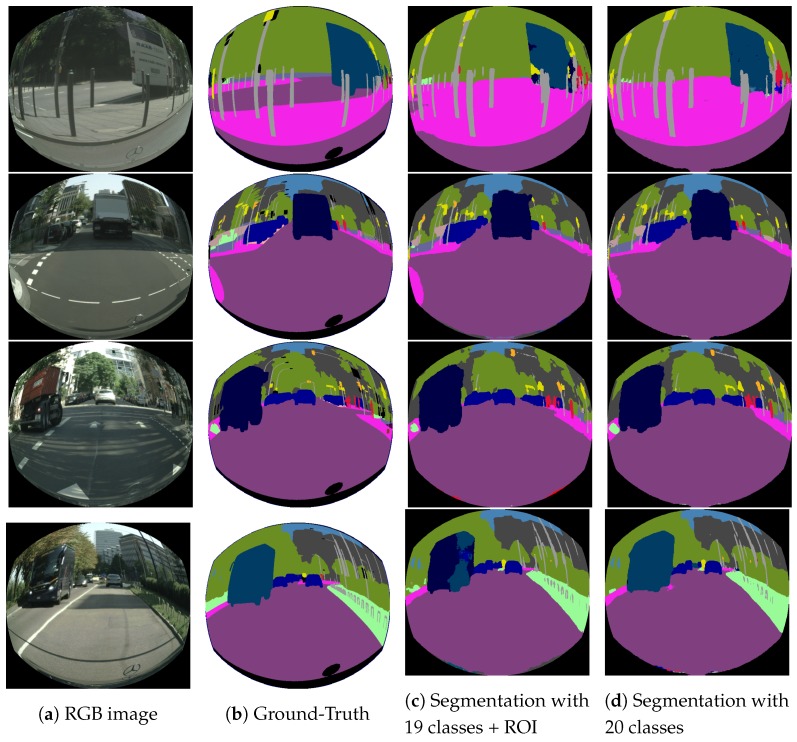
Comparison between different training strategies.

Table 3 shows in the second column the mean class IoU% obtained by the different networks for an image resolution of 640 × 576. In the third column, the forward time in seconds using a single GTX 1080Ti is depicted. A different image resolution of 814 × 512 was adopted for this column in order to compare results with other works of the literature. Results show that the best IoU is achieved by the ERFNetPSP model with 61.6% outperforming the best previous score for this distortion level (RDCNet) by 3.7%. ERFNet achieves a similar score (61.5%) for 19 classes, rising to 62.2% for the 20 classes version (ERFNet20), due to the good segmentation results in the border areas (black zones in the image). In this last case, training improves the detection of the classes with fewer training samples that appear close to the borders as shown in Figure 7. Our ERFNet proposals obtain a higher score than for the rest of CNNs. From the re-implemented group of architectures, only PSPNet (59.2%) improves the RDCNet performance. The DRNet-D-54 achieves a similar score (57.6%) and SegNet clearly has worse performance (50.1%).

The modified MXNet ERFNet presents a poor performance (55.1%), but it is improved by the addition of factorized restricted deformable convolutions (56.1%) and restricted deformable convolutions (57.9%). As shown in Table 3, regarding processing time, ERFNet MX is the fastest architecture, needing only 0.016 s to process a 814 × 512 image and achieving more than 63 fps. The second fastest is RDCNet with 0.018 s and 55 fps, followed by the original ERFNet (50 fps) and ERFNetPSP (>45 fps). From the rest of the tested networks, only SegNet works in real time (>14 fps) and PSPNet and DRN-D-54 have low frame ratings (4 and 6 fps).

**Figure 8:**
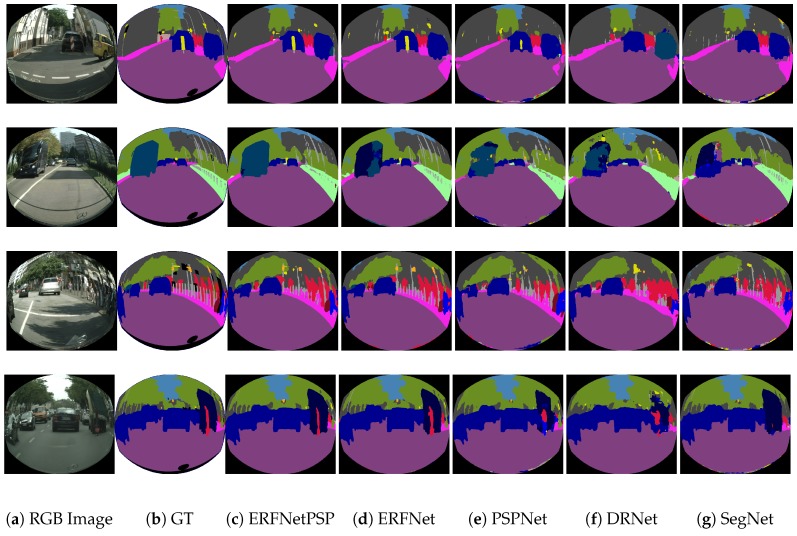
Qualitative results for different tested network models.

Table 4 shows detailed per-class results for the tested networks on the 640 × 576 dataset. As it can be seen, most of the best per-class scores are achieved by the ERFNet architectures. However, ERFNetPSP obtains the best results due to its outstanding performance in classes with few samples during training. Qualitative results for this table are presented in Figure 8.

## 6. Application to a Real Fisheye Camera

This experiment aims to demonstrate the generalization abilities provided by the suggested architectures and training techniques based on synthetic images obtained by using distortion models over normal FOV images, applying them to the images captured by a real fisheye camera, over an urban driving scenario similar to the one used during training, but never seen before. With that purpose, a HD fisheye camera with a 180${}^{\circ }$ FOV and a 1920 × 1080 resolution (USBFHD01M-BL180), manufactured by ELP, was used to record a set of sequences in the Campus of the University of Alcala (Spain) using our open-source autonomous electric car.

The previous training was not adequate for the real fisheye camera, due to the difference between resolutions and aspect-ratio of the synthetic images regarding the real ones. A new specific training adapted to the real fisheye camera was carried out, using nine new images with random distortions between $f_{inf}=200$ and $f_{sup}=700$, and a new one with $f_{c}=500$ with resolutions of 1120 × 792, in order to preserve a similar aspect-ratio to the real fisheye camera (1536 × 1080 without borders).

Both ERFNet and ERFNetPSP architectures were trained using the new range of distortions and resolution. A quantitative validation was performed using the transformed CityScapes validation subset for these new parameters. Once again, the ERFNetPSP reaches the best performance, obtaining a mean IoU of 69.6% while the baseline ERFNet achieves an IoU of 68.3% for this validation subset.

Table 5 shows the frame-rate achieved by the introduced architectures using a single GTX 1080Ti for 1536 × 1080 images.

**Figure 9:**
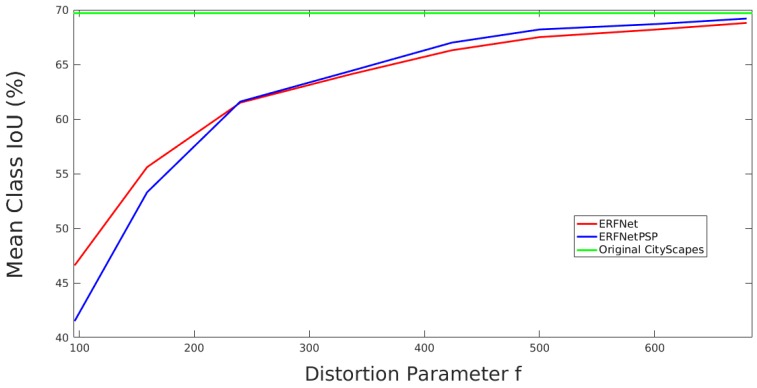
Mean class IoU performance vs. level of added distortion for basic training.

Figure 9 analyzes the performance of the two architectures on the validation subset as a function of distortion without using any data augmentation. As we can see, ERFNetPSP achieves better performance than ERFNet for medium and high distortions. For the strongest distortions, which damage context information, the behaviour is the opposite. Performance of both architectures improve as the added distortion is reduced and becomes similar to the performance of ERFNet on the original CityScapes dataset.

**Figure 10:**
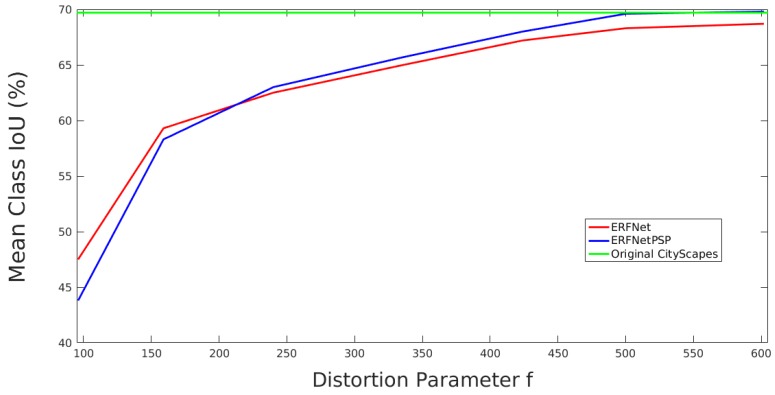
Results for validation subset with training with full data augmentation.

Figure 10 depicts a similar analysis but includes a pre-trained model on ImageNet and full data augmentation (random distortions and geometric and color transformations) in the training. As it can be seen, performance of both CNNs is clearly better, obtaining the best results with the ERFNetPSP architecture for light and medium distortions and with the baseline network for strong distortions. From the analysis of the real camera, an approximate value of 350 is estimated for the parameter *f*. Therefore, following the graphics, ERFNetPSP with ImageNet pre-training and full data augmentation obtains the best semantic segmentation results.

**Figure 11:**
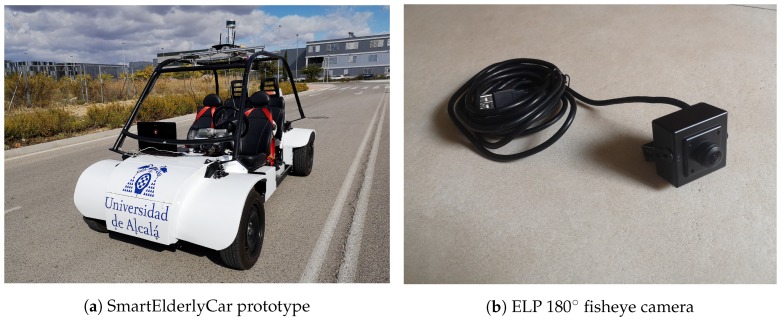
Autonomous open-source electric car and fisheye camera.

Fisheye camera was integrated in an open-source autonomous car prototype [33,34] as a complement to its main perception system, formed by a ZED camera, manufactured by StereoLabs, a VLP-16 LiDAR, manufactured by Velodyne, a HiPer Pro RTK-GPS receiver by TOPCON and odometry sensors by Kubler. Environment perception of the prototype is based on 3D semantic segmentation obtained from the fusion of LiDAR and segmented images, which is able to detect obstacles in a 3D environment [35]. Semantic segmentation for the fisheye camera runs on an embedded Jetson TX2 GPUs, manufactured by NVIDIA, and reaches 10 fps, which is the acquisition frequency of the rest of the sensors. Figure 11 shows the electric prototype and the camera used during the tests.

**Figure 12:**
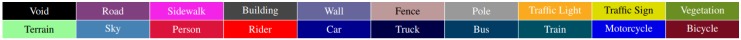
Cityscapes color legend.

Due to the absence of annotated ground-truth, only qualitative results are exposed in this section. To provide a convincing validation, results are split focusing on the main groups of segmented classes defined in Cityscapes, and using some representative Campus images captured from the autonomous vehicle. To facilitate the understanding of the segmentation, we provide the Cityscapes color legend in Figure 12.

**Figure 13:**
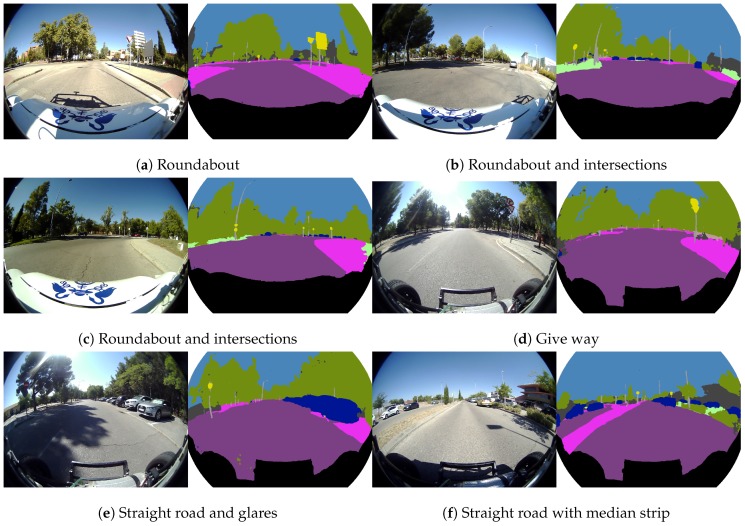
Real fisheye camera semantic segmentation examples for flat group.

Figure 13 illustrates various complex situations focused on the “flat group”, mainly composed of road and sidewalk classes, where the wider FOV of fisheye cameras clearly improves the scene comprehension about driving areas achieved with traditional cameras. Different images including roundabouts, intersections, pedestrian crosswalks and give-ways are depicted, where the road and the sidewalk classes are correctly segmented even in glare images and with obstacles, which helps to delimit the areas where the vehicle can drive in an autonomous way. The wide FOV of this camera provides more information about the lateral zones of the vehicle, which is vital in order to perform turning maneuvers in a safe way.

**Figure 14:**
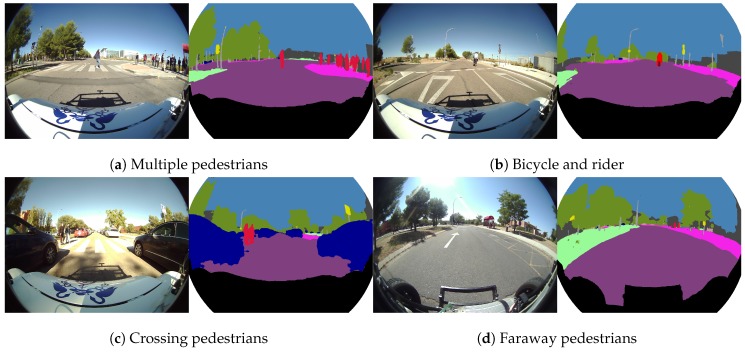
Real fisheye camera semantic segmentation examples for the human group.

Figure 14 shows some representative examples for the “human group” segmentation (person and rider classes), which is very important to correctly detect vulnerable users and avoid accidents. Figure 14a shows how fisheye cameras help to handle dynamic crosswalks, where many pedestrians on the sidewalks and on the road are detected at the same time with just one camera, providing excellent scene-understanding. On the left side of Figure 14b, we can find a bicycle and a rider correctly segmented and, in Figure 14c,d, different pedestrians segmented at short and long distances, respectively.

**Figure 15:**
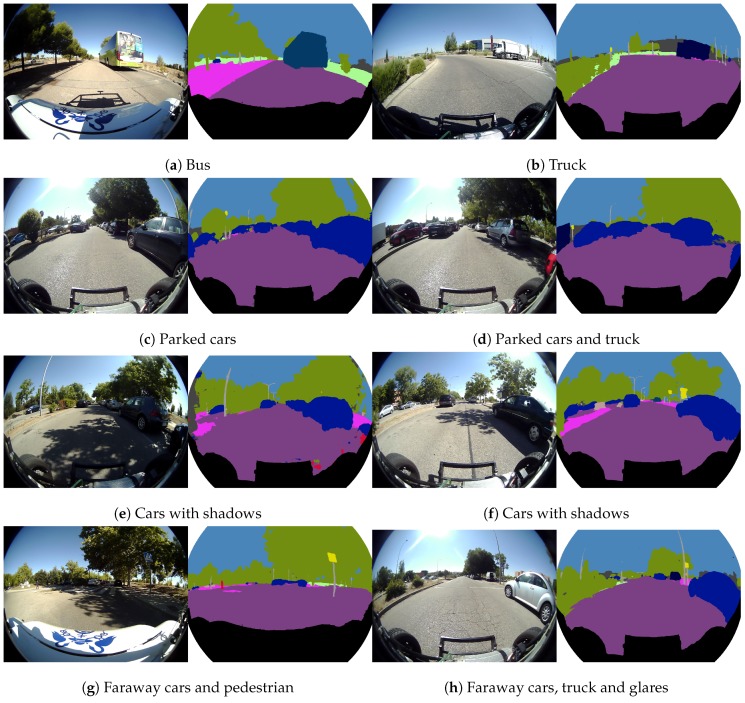
Real fisheye camera semantic segmentation examples for the vehicle group.

Figure 15 depicts some examples for the “vehicle group” segmentation, which includes car, truck, bus, motorcycle and bicycle classes. Figure 15a shows a case of segmented bus and Figure 15b a segmented truck. Figure 15c,d illustrate the segmentation of many cars parked in both sides of the road. Figure 15e,f show cars segmented under hard shades, and Figure 15g,h a couple of cases of long distance segmented cars.

**Figure 16:**
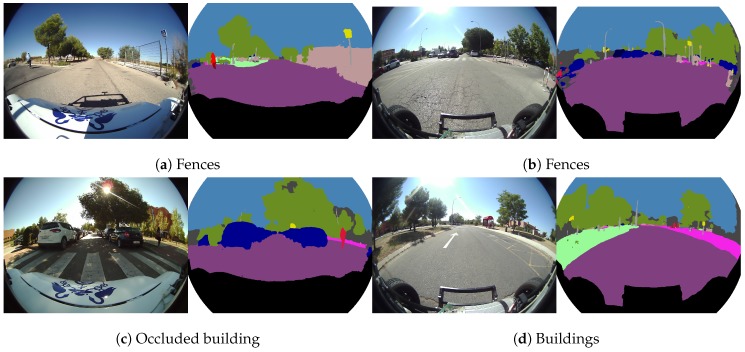
Real fisheye camera semantic segmentation examples for the construction group.

Figure 16 illustrates some segmentation cases focused on the “construction group” (building and fence classes). On the right side of Figure 16a,b, the segmentation of different fences are depicted, and Figure 16c,d show a couple of images with many segmented buildings.

**Figure 17:**
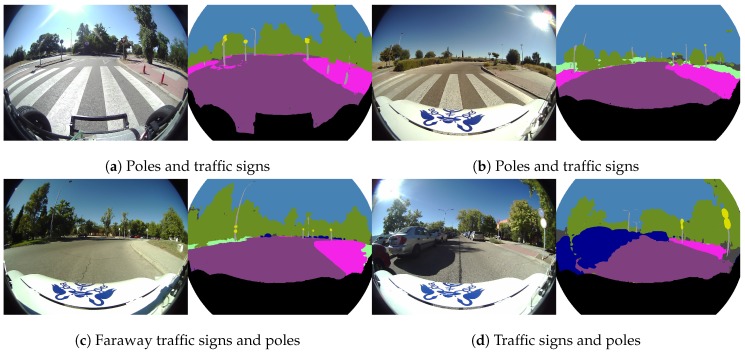
Real fisheye camera semantic segmentation examples for the object group.

The “object group” segmentation, composed of the following classes: pole, traffic light and traffic signs, is shown in Figure 17. These images show many cases of correct segmentation for pole and traffic sign classes. These objects are usually very small in the image and less frequent than other classes, which is derived from a few pieces of training data and therefore a more difficult segmentation.

Segmentation of the “nature group”, which includes vegetation and terrain classes, and the “sky group”, which only contains the sky class, are well represented in all of the previous images. Their influence is secondary in autonomous vehicles’ applications mainly due to the fact that they are faraway from the driving area. However, there are some cases where nature classes define the limits of the road (Figure 14d or Figure 15a) and should be taken into account.

Results demonstrate that the ERFNetPSP architecture provides real-time good quality semantic segmentation being able to detect even the classes with reduced number of training data and showing a robust behaviour to shadows and lighting changes.

**Figure 18:**
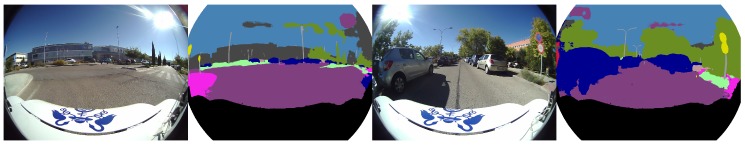
Problems with sky segmentation and glares.

Despite the good results, segmentation has still some problems dealing with glares (which are common in fisheye cameras due to its wide FOV) and with big classes with changing appearances such as the sky, as we can see in Figure 18.

An additional problem is that appearances of the classes present near the edges, corresponding to lateral objects located on the left/right FOV limits, are not included in the training dataset, which is captured from a conventional FOV camera. This fact degrades the obtained segmentation in the edge regions, which tend to associate small classes with more available classes such as building, road or sky.

## 7. Conclusions

This paper proposed a methodology to achieve real-time semantic segmentation based on ERFNet over real fisheye images, leveraging only synthetic images and, therefore, solving the lack of large-scale fisheye datasets while avoiding the heavy task of data annotation. The two introduced architectures (ERFNet and ERFNetPSP) achieve better results than the best state-of-the-art works in various synthetic datasets. Furthermore, the ability of ERFNetPSP to handle distortion by the prioritization of context information is proven, showing a better performance than ERFNet. The model also demonstrates better leveraging our data augmentation strategy, reaching the ERFNet performance in the original CityScapes dataset. Additionally, alternative training with an extra class to segment the image borders is presented. Our proposals have been validated with images taken from a real fisheye camera in unseen scenarios, showing a high capacity of domain adaptation without using a fine-tuning process with manually annotated data.

Future work involves the development of a full 360${}^{\circ }$ vision system based on three fisheye cameras and the following fusion with the LiDAR sensor data, in order to develop a complete 3D surrounding perception system. In addition, we plan to research methods to improve segmentation of the small classes close to the fisheye image borders.

## Figures and Tables

**Table 1:** Layer disposal of our proposed architecture.

Layer	Type	Out-F	Out-Re
1	Down-sampler block	16	320 × 288
2	Down-sampler block	64	160 × 144
3–7	5 × Non-bt-1D	64	160 × 144
8	Down-sampler block	128	80 × 72
9	Non-bt-1D (dilated 2)	128	80 × 72
10	Non-bt-1D (dilated 2)	128	80 × 72
11	Non-bt-1D (dilated 4)	128	80 × 72
12	Non-bt-1D (dilated 8)	128	80 × 72
13	Non-bt-1D (dilated 16)	128	80 × 72
14	Non-bt-1D (dilated 2)	128	80 × 72
15	Non-bt-1D (dilated 4)	128	80 × 72
16	Non-bt-1D (dilated 8)	128	80 × 72
17	Non-bt-1D (dilated 2)	128	80 × 72
18a	Layer 17 feature map	128	80 × 72
18b	Pooling and Convolution	32	80 × 72
18c	Pooling and Convolution	32	40 × 36
18d	Pooling and Convolution	32	20 × 18
18e	Pooling and Convolution	32	10 × 9
18	Up-Sampler and Concatenation	256	80 × 72
19	Convolution	C	80 × 72
20	Up-Sampler	C	640 × 576

**Table 2:** Architectures and data augmentation performances.

Network	Data Augmentation	Class IoU (%)
Dilation10 [26]	None	51.7
ResNet-26 [17]	None	52.0
OPPNet [17]	None	52.6
	**Fixed z-aug**	**54.5**
ERFNetPSP	None	53.3
	**Pretrained**	55.5
	**Fixed z-aug**	55.9
	**Random z-aug**	56.2
	**Full & pretrained**	**58.3**
ERFNet [28]	None	55.6
	**Pretrained**	56.3
	**Random z-aug**	56.8
	**Fixed z-aug**	57.0
	**Full & pretrained**	**59.3**

**Table 3:** Performance-efficiency comparison for the presented architectures.

Architecture	Class IoU (%)	Forward Pass Time (s)
	(640 × 576)	(814 × 512)
ERFNetPSP	**61.6**	0.022
ERFNet [28]	61.5	0.020
ERFNet20	**62.2**	0.020
PSPNet [31]	59.2	0.22
RDCNet [18]	57.9	0.018
DRN-D-54 [32]	57.6	0.146
FRDCNet [18]	56.1	-
ERFNet MX [18]	55.1	**0.016**
SegNet [30]	50.1	0.07

**Table 4:** Per-class IoU (%) on the fisheye CityScapes validation set compared to similar works.

Network	Roa	Sid	Bui	Wal	Fen	Pol	TLi	TSi	Veg	Ter	Sky	Ped	Rid	Car	Tru	Bus	Tra	Mot	Bic	IoU
**SegNet**	96.8	65.1	79.6	25.7	19.9	29.6	29.1	36.6	84.3	42.9	89.3	58.4	29.1	85.3	37.6	48.9	17.6	25.8	49.8	50.1
**DRNet**	97.0	67.7	82.4	34.6	31.4	30.3	36.5	49.6	85.4	47.3	88.9	66.4	43.3	87.2	51.3	65.5	35.4	36.0	57.4	57.6
**PSPNet**	97.2	68.7	83.2	34.9	31.3	31.4	37.5	48.7	85.7	47.7	89.5	66.7	44.6	88.3	57.4	67.7	**45.0**	40.1	59.1	59.2
**ERFNet 20**	97.4	70.2	83.8	34.7	31.6	**40.7**	42.2	55.2	**87.1**	52.4	89.5	69.1	47.9	88.7	52.3	69.4	30.5	40.9	60.1	60.3
**ERFNet**	**97.4**	**70.8**	**83.9**	**37.2**	29.4	39.2	41.9	**55.3**	86.8	**53.2**	89.8	**69.7**	**49.4**	**89.1**	56.2	**76.1**	42.3	**41.4**	59.9	61.5
**ERFNetPSP**	97.3	70.1	83.2	35.9	**31.8**	37.2	**42.3**	54.7	86.3	52.3	**90.4**	68.7	48.2	88.8	**64.2**	74.2	41.6	41.1	**60.2**	**61.7**

**Table 5:** Forwarding time and frame-rate for the presented architectures.

Architecture	Forward Pass Time (s)	Frames per Second
	(1536 × 1080)	
ERFNetPSP	0.088	11.4
ERFNet [28]	0.081	**12.3**
